# Earthworm Grazed-*Trichoderma harzianum* Biofortified Spent Mushroom Substrates Modulate Accumulation of Natural Antioxidants and Bio-Fortification of Mineral Nutrients in Tomato

**DOI:** 10.3389/fpls.2018.01017

**Published:** 2018-07-17

**Authors:** Udai B. Singh, Deepti Malviya, Wasiullah Khan, Shailendra Singh, N. Karthikeyan, Mohd. Imran, Jai P. Rai, B. K. Sarma, M. C. Manna, Rajan Chaurasia, Arun K. Sharma, Diby Paul, Jae-Wook Oh

**Affiliations:** ^1^Plant-Microbe Interaction and Rhizosphere Biology Lab, ICAR-National Bureau of Agriculturally Important Microorganisms (ICAR-NBAIM), Maunath Bhanjan, India; ^2^Department of Biosciences, Faculty of Science, Integral University, Lucknow, India; ^3^Department of Mycology and Plant Pathology (Krishi Vigyan Kendra), Institute of Agricultural Sciences, Banaras Hindu University, Varanasi, India; ^4^Department of Mycology and Plant Pathology, Institute of Agricultural Sciences, Banaras Hindu University, Varanasi, India; ^5^Division of Soil Biology, ICAR-Indian Institute of Soil Science, Bhopal, India; ^6^Department of Botany, Faculty of Science, Banaras Hindu University, Varanasi, India; ^7^Pilgram Marpeck School of STEM, Truett McConnel University, Cleveland, GA, United States; ^8^Department of Animal Biotechnology, Konkuk University, Seoul, South Korea

**Keywords:** *Trichoderma harzianum*, solid-state cultivation, spent mushroom substrate, antioxidants, ROS, lipid peroxidation, protein oxidation

## Abstract

The present investigation was aimed at evaluating the impact of earthworm grazed and *Trichoderma harzianum* biofortified spent mushroom substrate (SMS) on natural antioxidant and nutritional properties of tomato. Results of the investigation reveal that earthworm grazing and *T. harzianum* bio-fortification led to significant improvement in the physico-chemical properties of fresh SMS and its application increased the accumulation of natural antioxidants and mineral content in tomato as compared to either *T. harzianum* biofortified SMS or fresh SMS. In particular, the earthworm grazed, *T. harzianum* biofortified SMS (EGTHB-SMS) was found to inhibit lipid peroxidation and protein oxidation with significant increase in total polyphenol and flavonoid content in tomato. Further, it increased Fe^2+^/Fe^3+^ chelating activity, superoxide anion radical scavenging activity compared to other treatments. The results thus suggest an augmented elicitation of natural antioxidant properties in tomato treated with EGTHB-SMS, resulting in a higher radical scavenging activity, that is highly desirable for human health. In addition, the use of SMS to enhance the nutritional value of tomato fruits becomes an environment friendly approach in sustainable crop production.

## Introduction

People are increasingly becoming more conscious about their health and more attention is being paid to the nutritional value of human diet with an eye on role of mineral nutrients and antioxidants in health. Several epidemiological studies have reported that high intake of green vegetables, fruits and pulses is associated with reduced risk of a number of chronic diseases (Singh et al., 2010) due to imbalance in the antioxidant and prooxidant homeostasis in human body (Nautiyal et al., 2008). Such conditions dominate either due to increased generation of free radicals (FRs) caused by excessive oxidative stress or due to poor scavenging/quenching of FRs in the body (Bhanja et al., 2009; Ahmad et al., 2016). Free radicals are chemically unstable compounds that results in damage to lipid cells, proteins and DNA (Ahmad et al., 2016). Reactive oxygen species (ROS) are produced intra-cellularly through multiple mechanisms and depending on the cell and tissue types, the major sources being the ‘professional’ producers of ROS NADPH oxidase (NOX) complexes in cell membranes, mitochondria, peroxisomes, and endoplasmic reticulum (Singh et al., 2009b; Singh et al., 2010). Biological systems have several antioxidant defense mechanisms that prevent the destructive effects of ROS and FRs such as antioxidative compounds and enzymes (Nautiyal et al., 2008; Ahmad et al., 2016). In aerobic organisms, the major target sites for ROS and FRs are the cellular membrane lipids, proteins and DNA and they cause alterations in membrane structure and its function (Ahmad et al., 2016). A wide range of antioxidants, both natural and synthetic have been used as external supplements in the regular diet that could influence and suppress/slow down the processes including lipid oxidation, binding of transitional metal ion catalysts, decomposition of peroxides, etc. Increase in FR scavenging through simple or complex cellular mechanisms both in living organisms as well as in the food industry has been well documented (Prakash et al., 2007b; Singh et al., 2009b, 2010). Due to the apparent toxicity of synthetic antioxidants, currently, the interest of the society in natural antioxidants is increasing day by day (Huang and Wang, 2004).

Tomato (*Solanum* *lycopersicum*) is one of the most important vegetable crops consumed across the various climatic regions of the world. Tomato and its products serve as rich sources of dietary bioactive molecules such as lycopene, beta-carotene, phytoene, phytofluene, folic acid, flavonoids and vitamin C (George et al., 2004; Hyman et al., 2004). These bioactive compounds are well-studied for their health protective abilities especially against cardiovascular diseases, coronary heart diseases and various types of cancer. Similarly, *Trichoderma harzianum* (avirulent, plant beneficial fungus) is a common soil inhabitant broadly distributed in terrestrial ecosystems (Harman et al., 2004) and plays many important roles in diverse natural environments. Although the fungus absorbs nutrition from dead organic materials as a saprophyte, majority of its population is concentrated in nutrient rich niches like the rhizosphere. In agriculture, *T. harzianum* is primarily known for its biocontrol activities against soilborne phytopathogens but other multifarious activities have also been recorded by various researchers. Therefore, the ability of the fungus to colonize roots of crop plants is of a great advantage when it is used as a nutrient mobilizer, plant growth promoter and biological control agent (Harman et al., 2004; Sarma et al., 2015). The ability to colonize root and endophytic behavior of *Trichoderma* spp. in plant roots are proven and it was found that *Trichoderma* responds chemotropically to roots. The response of root cells to colonization by fungi may have profound implications in the performance of these organisms as plant growth promoting as well as biocontrol agents of soilborne plant pathogens (Harman et al., 2004). There are strains of *T. harzianum* which improve nutrient uptake and translocation in plants, resulting in enhanced nutritional value of the produce there from Glick (1995), Berg (2009).

Spent mushroom substrate (SMS) is a by-product of mushroom industry, rich in organic matter and other essential nutrients for plant growth. Annually, mushroom industries release more than 50 million tons of SMS as waste material (Fox and Chorover, 1999) which is vastly under utilized. A larger portion of SMS is carelessly disposed outside the production unit itself begetting a number of environmental issues. Piling-up of SMS may cause various environmental problems/pollution which further leads to groundwater contamination and production of greenhouse gasses such as carbon dioxide and nitrous oxide causing global warming and loss of essential plant nutrients (Beyer, 1996). In recent years, attention has been made toward more amicable ways of SMS disposal. These surplus residues can be recycled for sustaining soil health and enhancing crop productivity because the SMS is rich source of organic matter and mineral nutrients. Some of the researchers used SMS as organic amendment in various vegetable and high value crops and reported positive effect on crop yield (Beyer, 1996; Ahlawat et al., 2006). Earthworm grazing is a biooxidation process accelerating the stabilization of organic matter involving the joint action with microorganisms which are responsible for the physico-biochemical degradation of organic matter present in the waste. Earthworms are the important drivers of the process, conditioning the substrate and altering physico-biological activities (Aira et al., 2002; Domınguez, 2004). Further, earthworm has indirect effects on the structure and activities of microbial communities present in the waste through stimulation of microbial populations, inoculum dispersal, litter comminution, grazing, gut passage and aggregate formation (Anderson, 1987; Aira et al., 2002). Earthworms are involved in the fragmentation and ingestion of fresh organic matter, providing a greater surface area for the microbial colonization, and thereby drastically altering biological activity (Lores et al., 2006). Earthworms also have a great impact on nutrient mineralization and transformation during vermicomposting through modifications of the environmental conditions in the organic wastes and their interactions with microbes which favor the nitrogen transformation by rapid conversion of ammonia-nitrogen into nitrates (Aira et al., 2008; Domınguez et al., 2010). It increases the macro- and micronutrients, plant growth hormones auxins, gibberellins and cytokinins (Krishnamoorthy and Vajrabhiah, 1986), humic acids (Atiyeh et al., 2002) and enzymes in the decomposed organic manures (Sarma et al., 2010) which not only enhance plant growth but also hold nutrients for longer periods (Ndegwa and Thompson, 2001; Singh et al., 2013a). But so far no attempt seems to be made on value addition of SMS using *T. harzianum* and its synergistic effect with earthworm (*Eisenia fetida*). Moreover, the effect of earthworm grazed and *T. harzianum* bio-fortified SMS on nutritional quality and natural antioxidant properties of tomato is also an unexplored area. Recognizing the importance of nutritional value of tomato in terms of antioxidant properties and other mineral nutrients and their role in human health, the present study was undertaken with the objectives to study (1) the effect of earthworm grazing and *T. harzianum* bio-fortification on value addition of SMS, and (2) the impact of earthworm grazed and *T. harzianum* bio-fortified SMS on natural antioxidant and other nutritional properties of tomato.

## Materials and Methods

### Source of Reagents and Media

Culture media were procured from HiMedia, India, whereas, chemical reagents including standards were procured from Sigma–Aldrich, India. Bovine serum albumin (BSA), lycopene, β-carotene, organic solvents and other chemicals and analytical grade solvents were purchased from Merck Biosciences, India.

### Fungal Strain, Earthworm (*Eisenia fetida*) and Culture Condition

The test fungal strain, *T. harzianum* UBSTH-501 (GenBank Accession No: MG972984) was obtained from Plant-Microbe Interaction and Rhizosphere Biology Lab, ICAR-National Bureau of Agriculturally Important Microorganisms (ICAR-NBAIM), Kushmaur, Maunath Bhanjan, India and maintained on potato dextrose agar (PDA) by sub-culturing at 25 ± 2°C at 15 days interval. Earthworms (*E. fetida*) were obtained from Dr. M. C. Manna, Division of Soil Biology, ICAR-Indian Institute of Soil Science, Bhopal, India.

### *In Planta* Study

#### Experimental Design

The effects of earthworm grazed, *T. harzianum* biofortified SMS on nutritional quality of tomato were evaluated under nethouse conditions at ICAR-NBAIM, India. The treatments were: T_1_- Control (without SMS), T_2_- Fresh SMS, T_3_-earthworm grazed SMS, T_4_- *T. harzianum* biofortified SMS and T_5_- earthworm grazed, *T. harzianum* biofortified SMS. Experiments were arranged in a completely randomized block design under nethouse conditions and each treatment consisted of five replications.

#### Preparation of Earthworm Grazed and *T. harzianum* Bio-Fortified SMS

**FIGURE 1:**
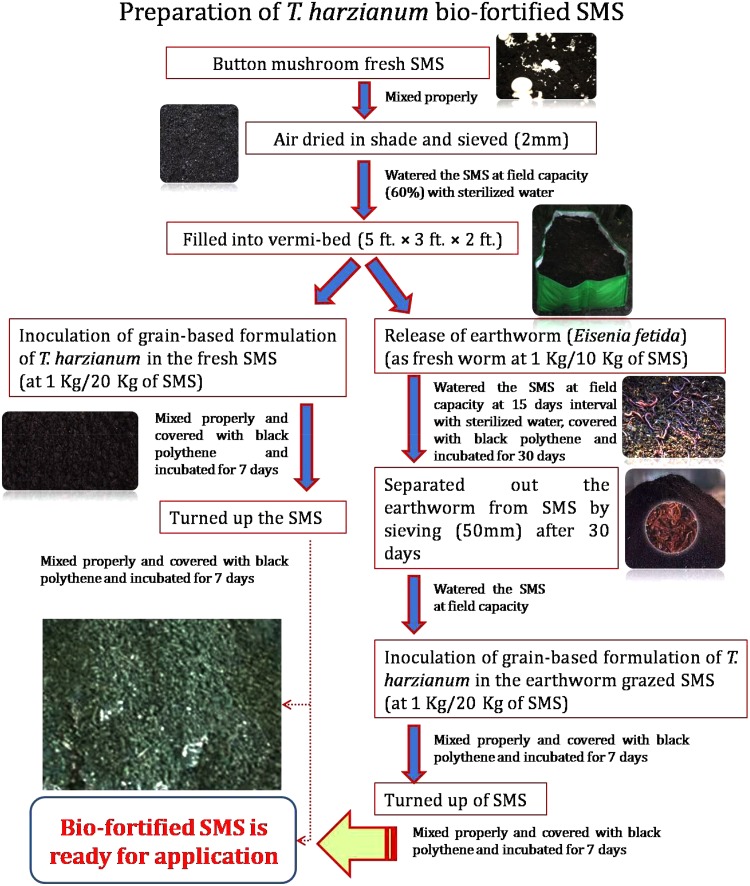
Flow diagram showing a newly developed technique for preparation of earthworm grazed and *Trichoderma harzianum* UBSTH-501 biofortified SMS.

Fresh SMS of white button mushroom (*Agaricus bisporus*) was collected from Department of Mycology and Plant Pathology, Institute of Agricultural Sciences, Banaras Hindu University, Varanasi, India. It was subsequently air-dried under shade, sieved (2 mm pore size) and filled into Vermi-bed of the dimensions of 5 × 3 × 2 ft. Grain-based bioformulation of *T. harzianum* was prepared following the procedure described by Singh et al. (2012). *T. harzianum* bio-fortified SMS was prepared as per the methods/steps described in **Figure 1**. Physico-chemical properties of fresh SMS, earthworm grazed SMS, *T. harzianum* biofortified SMS and earthworm grazed and *T. harzianum* biofortified SMS were analyzed. At the time of application, the population of *T. harzianum* in earthworm grazed and *T. harzianum* biofortified and only *T. harzianum* biofortified SMS were 3.20 × 10^6^ and 2.25 × 10^6^ cfu g^−1^, respectively.

#### Planting Material and Growth Condition

Tomato seedlings (*cv*. Dev, Nunhems Pvt. Ltd., India) were grown in sterile potting mixture adopting standard horticultural practices at ICAR-NBAIM, Maunath Bhanjan, India. Forty-five days old tomato plants were uprooted gently without much disturbance to the roots and transplanted into pots in nethouse. The experiments were conducted during November to February with the range of relative humidity being 80–85% under 11/13 h light/dark photoperiod.

#### Soil Collection, Preparation and Analysis

Soil was collected from an agricultural farm, Indian Institute of Seed Science (formerly known as Directorate of Seed Research), Kushmaur, Maunath Bhanjan, India. Such soil was further processed for the study by sieving (2 mm pore size) and air drying. After processing, the soil was mixed with vermicompost in a 4:1 ratio (w/w) and chemical fertilizers, *viz.* diammonium phosphate, urea and muriate of potash at 174, 326, and 100 kg per hectare, respectively, followed by its sterilization by autoclaving at 121°C for 60 min twice at 12 h interval and storage in the same position. The physico-chemical properties of experimental soil were analyzed after 5 days of sterilization (**Table 1**).

**Table 1:** Physico-chemical characteristics of initial experimental soil.

S.N.	Soil properties	Values
(1)	Textural class	Silt loam
(2)	pH	7.2
(3)	EC (dS m^−1^)	0.98
(4)	OC (g kg^−1^)	5.4
(5)	OM (g kg^−1^)	9.04
(6)	Bulk density (Mg m^−3^)	1.40
(7)	Particle density (Mg m^−3^)	2.50
(8)	CaCO_3_ (%)	6.55
(9)	**Available macronutrients (kg ha^−1^)**	
(i)	N	189.41
(ii)	P	29.70
(iii)	K	149.79
(iv)	S	8.65
(10)	**Available micronutrients (mg kg^−1^)**	
(i)	Fe	14.68
(ii)	Mn	2.50
(iii)	Cu	0.97
(iv)	Zn	0.64
(v)	B	0.11
(vi)	Mo	0.06

### Nethouse Study

Forty-five days old tomato plants were transplanted into pots each containing 200 g of fresh and bio-fortified SMS. The SMS was thoroughly mixed in the pots containing experimental soil (5 kg) and a single plant was transplanted into each pot. SMS free soil served as the negative control and soil with fresh SMS was taken as positive control. Further, two blocks were randomly arranged to replicate the five treatments and each treatment with five replicates (pots) was randomly arranged within each block.

### Estimation of Natural Antioxidants and Nutritional Quality of Ripe Tomato Fruits

Five ripe tomato fruits were randomly picked from each treatment for further analysis. The contents of total carbohydrate, soluble sugar, protein and lutein zeaxanthin were determined according to Sadasivam and Manickam (1996), whereas vitamin A (as retinal) and ascorbic acid were quantified according to Thimmaiah (2012). Lycopene and β-carotene content in ripe tomato fruits were measured according to Hyman et al. (2004). N content in the ripe fruits was quantified by Kjeldahl method, whereas the composition of minerals and micronutrients was determined according to Sahu (2011).

A modified thiobarbituric acid-reactive species (TBARS) assay of Ohkawa et al. (1979) was used to measure lipid peroxidation with slight modifications (Singh et al., 2010). Similarly, protein oxidation was estimated according to the methods of Singh et al. (2009b) with slight modifications (Singh et al., 2010). The contents of total polyphenols and flavonoids were determined according to Singh et al. (2009a). The ferrous and ferric ion chelating properties and superoxide anion radical scavenging ability of ripe tomato extract were determined according to Singh et al. (2010). β-carotene bleaching assay was performed by the auto-oxidation of β-carotene and linoleic acid coupled reaction method according to Singh et al. (2010). Peroxidase and catalase activities were measured according to Thimmaiah (2012). A unit of catalase is defined as the quantity of enzyme necessary to decompose 1 μM of H_2_O_2_ per minute at 25°C, whereas 1 unit of peroxidase is equal to 0.1 of absorbance. Superoxide dismutase activity of tomato extracts was determined following the method of Sadasivam and Manickam (1996). The effect of *T. harzianum* biofortified SMS on number of fruit per plants and yield was measured after final harvest of fruits. For this purpose, five plants were randomly selected from each treatments and first picking was done after 75 days of transplanting. A total of five picking was done at 10 days interval and finally average number of fruits per plants and yield (kg per plant) were calculated.

### Effect of Earthworm Grazed and *T. harzianum* Bio-Fortified SMS on Physico-Biochemical Properties of Soil

The effects of earthworm grazed and *T. harzianum* bio-fortified SMS on physico-biochemical properties of soil were analyzed at the end of the experiment. Soil samples (200 g) were collected from each treatment and stored at 4°C till analysis. Soil pH and EC were measured in 1:2.5 soil-water mixture. Soil organic carbon was measured by using modified Walkley-Black method. However, bulk density, particle density CaCO_3_, available micronutrients (N, P, K, and S) and available micronutrients (Fe, Mn, Cu, Zn, B, and Mo) in the soil samples were analyzed by following the standard procedures described by Sahu (2011).

### Statistical Analyses

*In vitro* laboratory experiments were performed in a completely randomized design and the nethouse experiments were conducted in a completely randomized block design. Laboratory experiments were repeated three times, whereas nethouse experiments were repeated twice in five replications each. Data were subjected to analysis of variance (ANOVA) and Duncan’s Multiple Range Test using SPSS software Version 16.0 program. Data were compared with DMRT at *p*≤ 0.05. Graphs and figures were drawn using the statistical package Origin Version 8.0.

## Results

### Effect of Earthworm Grazing and *T. harzianum* Bio-Fortification on Nutritional Value of SMS

Results showed that earthworm grazing and *T. harzianum* bio-fortification alone or in combination significantly increased the biochemical and nutritional properties of fresh SMS (**Table 2**). The physico-chemical properties in terms of pH, EC, bulk and particle density showed significant improvement in EGTHB-SMS and only *T. harzianum* biofortified SMS (THB-SMS) over the earthworm grazed SMS (EG-SMS) and fresh SMS. However, marginal increase was recorded with respect to nitrogen content, whereas phosphorus and potassium content were significantly higher in EGTHB-SMS (1.79 and 285.50, respectively) over only THB-SMS (1.65 and 256.25, respectively), EG-SMS (1.25 and 241.50, respectively) and fresh SMS (1.18 and 231.0, respectively). Similar trend was recorded in case of calcium content (**Table 2**). Further, results of the investigation reveal the fact that *T. harzianum* biofortification and earthworm grazing significantly decrease the sodium, chloride and nitrate content, and simultaneously increased porosity and moisture content in the biofortified SMS as compared to other treatments (**Table 2**).

**Table 2:** Effect of solid-state cultivation of *Trichoderma harzianum* UBSTH-501 and earthworm (*Eisenia fetida*) grazing on physico-chemical properties of spent button mushroom substrate.

S.N.	Parameters tested	Fresh SMS	*E. fetida* grazed SMS	*T. harzianum* UBSTH-501 fortified SMS	*T. harzianum UBSTH-501* fortified and *E. fetida* grazed SMS
(1)	pH	8.4^a^	8.1^b^	7.8^c^	7.2^d^
(2)	EC	5.50^a^	4.15^b^	4.02^b^	3.21^c^
(3)	Organic carbon (%)	4.90^b^	4.95^b^	4.50^c^	5.20^a^
(4)	Nitrogen (%)	2.71^c^	2.75^c^	2.85^b^	2.96^a^
(5)	Phosphorus (%)	1.18^d^	1.25^c^	1.65^b^	1.79^a^
(6)	Potassium (ppm)	231.0^d^	241.50^c^	256.25^b^	285.50^a^
(7)	Calcium (ppm)	543.33^d^	552.35^c^	600.05^b^	660.50^a^
(8)	Sodium (ppm)	260.25^a^	260.12^a^	210.45^b^	180.00^c^
(9)	Chloride (ppm)	146.10^a^	120.25^b^	75.04^c^	60.66^d^
(10)	Nitrate (%)	12.80^a^	10.33^b^	8.96^c^	8.02^c^
(11)	Total dissolved solid (ppm)	1910.00^a^	1450.50^b^	1469.60^b^	1262.55^c^
(12)	Bulk density (g cm^3^)	0.57^a^	0.50^a^	0.40^b^	0.44^b^
(13)	Particle density (g cm^3^)	2.20^a^	2.10^a^	1.84^b^	1.75^b^
(14)	Porosity (%)	20.00^d^	24.25^c^	30.00^b^	39.60^a^
(15)	Moisture (%)	59.00^c^	62.33^b^	65.50^a^	66.90^a^

*EC represents Electrical Conductivity, ppm- part per million, cm-centimeter, data are means (n = 3), data with different letters show significant difference in row data in Complete Randomized Design at p < 0.05 under Duncan’s multiple range test.*

### Effect on Accumulation of Total Carbohydrate, Soluble Sugar, Proteins and Lutein Zeaxanthin Content

**FIGURE 2:**
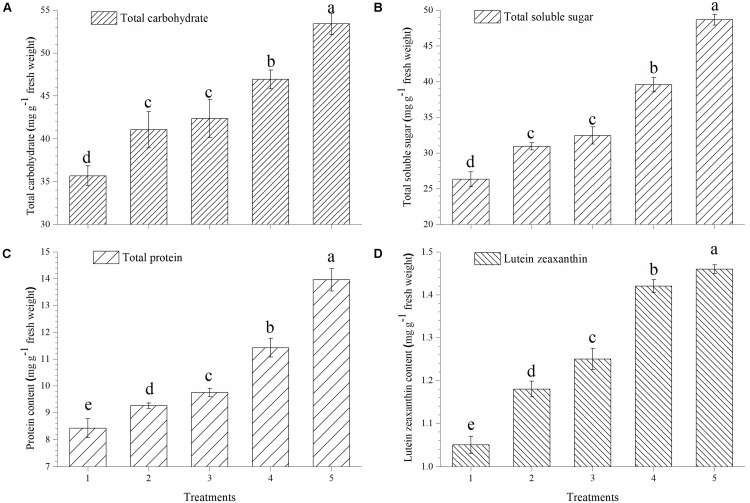
Effect of solid state cultivated *T. harzianum* UBSTH-501 on nutritional quality of tomato at harvest, **(A)** total carbohydrate, **(B)** total soluble sugar, **(C)** protein content, and **(D)** lutein zeaxanthin content. Treatments: 1- Control (untreated), 2- Fresh SMS, 3- Earthworm grazed SMS, 4- *T. harzianum* UBSTH-501 biofortified SMS, and 5- Earthworm grazed and *T. harzianum* UBSTH-501 biofortified SMS. Data are means (*n* = 5) and vertical bars represent standard error of means. Different letters above columns show significant difference in randomized block design test at *p* < 0.05 under Duncan’s multiple range test.

Plants treated with *T. harzianum* bio-fortified SMS showed higher accumulation of total carbohydrate, soluble sugar, proteins and lutein zeaxanthin in the ripe tomato fruits. The accumulation of total carbohydrate was significantly higher in the tomato fruits obtained from the plants treated with EGTHB-SMS (53.90 μg g^−1^ fresh wt.) when compared to only THB-SMS (46.92 μg g^−1^ fresh wt.), EG-SMS (42.35 μg g^−1^ fresh wt.), fresh SMS treated (41.04 μg g^−1^ fresh wt.) and control plants (35.67 μg g^−1^ fresh wt.) (**Figure 2A**). Similarly, significant increase in total soluble sugar content in ripe fruits was recorded in plants treated with EGTHB-SMS (48.67 μg g^−1^ fresh wt.) followed by only THB-SMS (39.56 μg g^−1^ fresh wt.), EG-SMS (32.45 μg g^−1^ fresh wt.), fresh SMS (30.92 μg g^−1^ fresh wt.) compared to the untreated control plants (26.33 μg g^−1^ fresh wt.) under nethouse conditions (**Figure 2B**). Similarly, the total protein content (**Figure 2C**) and lutein zeaxanthin content (**Figure 2D**) in ripe fresh tomato fruits were also significantly higher when treated with EGTHB-SMS compared to the other treatments under investigation.

### Effect on Accumulation of Vitamin A, β-Carotene, Lycopene and Ascorbic Acid

**FIGURE 3:**
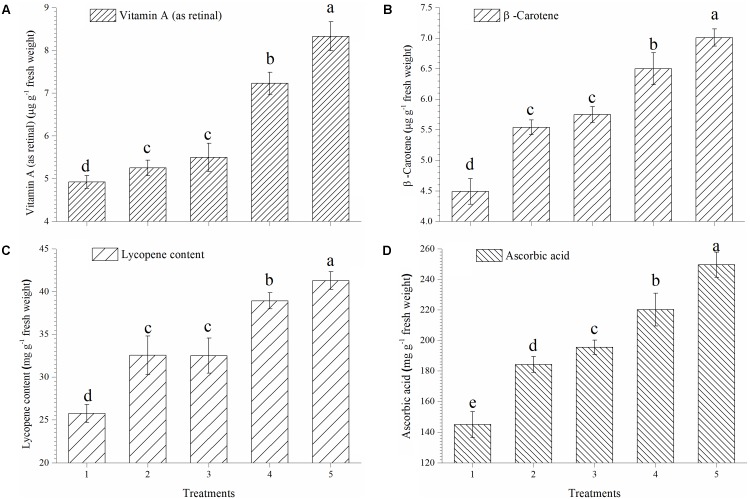
Effect of solid state cultivated *T. harzianum* UBSTH-501 on antioxidant compound in ripe tomato fruits at harvest. **(A)** Vitamin A (as retinal), **(B)** β-carotene content, **(C)** lycopene content, and **(D)** ascorbic acid content. Treatments: 1- Control (untreated), 2- Fresh SMS, 3- Earthworm grazed SMS, 4- *T. harzianum* UBSTH-501 biofortified SMS, and 5- Earthworm grazed and *T. harzianum* UBSTH-501 biofortified SMS. Data are means (*n* = 5) and vertical bars represent standard error of means. Different letters above columns show significant difference in randomized block design test at *p* < 0.05 under Duncan’s multiple range test.

In the present study, EGTHB-SMS recorded considerable increase in synthesis and accumulation of vitamin A, β-carotene, lycopene and ascorbic acid in ripe tomato fruits under nethouse experiments. Quantitative estimation showed that vitamin A in tomato fruits was significantly higher in the plants inoculated with EGTHB-SMS (8.33 μg g^−1^ fresh wt.) compared to other treatments (**Figure 3A**). Similarly, the highest β-carotene content was also recorded in tomato treated with EGTHB-SMS (7.01 μg g^−1^ fresh wt.) compared to only THB-SMS treated (6.50 μg g^−1^ fresh wt.), EG-SMS (5.75 μg g^−1^ fresh wt.), fresh SMS (5.54 μg g^−1^ fresh wt.) and untreated control plants (4.49 μg g^−1^ fresh wt.) (**Figure 3B**). Further, more or less similar trend was observed with lycopene (**Figure 3C**) and ascorbic acid content in ripe tomato fruits (**Figure 3D**).

### Effect on Accumulation of Mineral Content in Ripe Tomato Fruits

**FIGURE 4:**
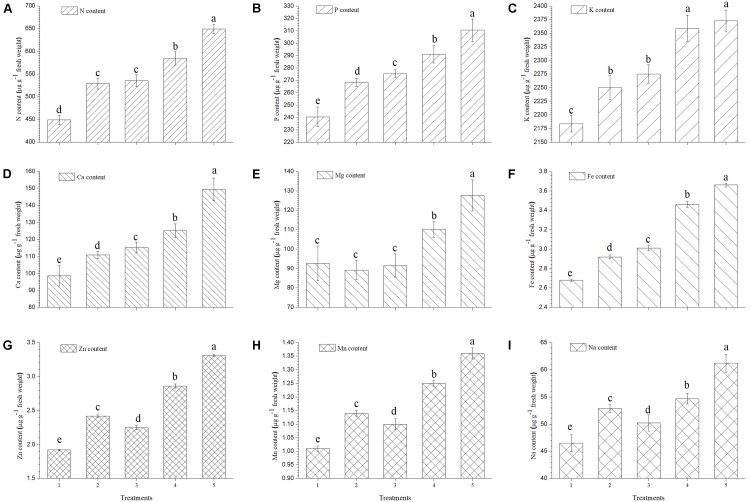
Effect of solid state cultivated *T. harzianum* UBSTH-501 on mineral content in ripe tomato fruits at harvest. **(A)** N content, **(B)** P content, **(C)** K content, **(D)** Ca content, **(E)** Mg content, **(F)** Fe content, **(G)** Zn content, **(H)** Mn content, and **(I)** Na content. Treatments: 1- Control (untreated), 2- Fresh SMS, 3- Earthworm grazed SMS, 4- *T. harzianum* UBSTH-501 biofortified SMS, and 5- Earthworm grazed and *T. harzianum* UBSTH-501 biofortified SMS. Data are means (*n* = 5) and vertical bars represent standard error of means. Different letters above columns show significant difference in randomized block design test at *p* < 0.05 under Duncan’s multiple range test.

The present study reveals a significant increase in mineral contents of ripe tomato fruits when inoculated with EGTHB-SMS compared to the only THB-SMS, EG-SMS, fresh SMS and control plants (**Figure 4**). Results of the investigation also establish that in ripe fruits from tomato plants inoculated with EGTHB-SMS induced 1.5–2 times more uptake and accumulation of nitrogen, phosphorus, potassium and calcium compared to those from plants treated with only THB-SMS, EG-SMS and fresh SMS (**Figures 4A–D**, respectively). Also, a similar trend was recorded for the magnesium and iron content of the ripe tomato fruits (**Figures 4E,F**). Again, maximum zinc, manganese and sodium content was recorded in tomato fruits from plants treated with EGTHB-SMS followed by only THB-SMS, fresh SMS and EG-SMS treated plants (**Figures 4G–I**, respectively), whereas minimum mineral content was recorded in control plants (**Figure 4**).

### Effect on Accumulation of Natural Antioxidants and Enzymatic Activities

In the present study, antioxidant activity was measured by different methods, namely presence of lipid peroxidation and protein oxidation assay, total polyphenols and flavonoids, ferrous and ferric ion chelating activity, superoxide anion radical scavenging activity, β-carotene bleaching assay and antioxidant enzymatic activity.

### Effect on Lipid Peroxidation and Protein Oxidation

**FIGURE 5:**
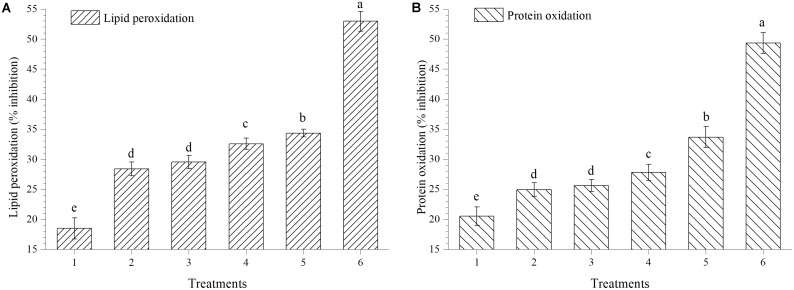
Effect of solid state cultivated *T. harzianum* UBSTH-501 on **(A)** lipid peroxidation and **(B)** protein oxidation in ripe tomato fruits at harvest. Treatments: 1- Control (untreated), 2- Fresh SMS, 3- Earthworm grazed SMS, 4- *T. harzianum* UBSTH-501 biofortified SMS, 5- Earthworm grazed and *T. harzianum* UBSTH-501 biofortified SMS and 6- Reference compound quercetin. Data are means (*n* = 5) and vertical bars represent standard error of means. Different letters above columns show significant difference in randomized block design test at *p* < 0.05 under Duncan’s multiple range test.

The activity of extracts to scavenge HO^⋅^ was also measured by protein oxidation method; extract obtained from plants treated with EGTHB-SMS inhibited the degree of lipid peroxidation and protein oxidation significantly (*p* < 0.01). Maximum inhibition in lipid peroxidation (34.34%) and protein oxidation (33.66%) was recorded in the fresh extract obtained from EGTHB-SMS treated plants followed by those treated with only THB-SMS, EG-SMS and fresh SMS treated plants at 10 mg ml^−1^ (**Figures 5A,B**). However, minimum inhibition of the mentioned processes was recorded in control plants. Further, the reference compound quercetin exhibited better inhibitory effects on lipid peroxidation and protein oxidation than other treatments (**Figure 5**).

### Total Polyphenol and Flavonoid Content

**FIGURE 6:**
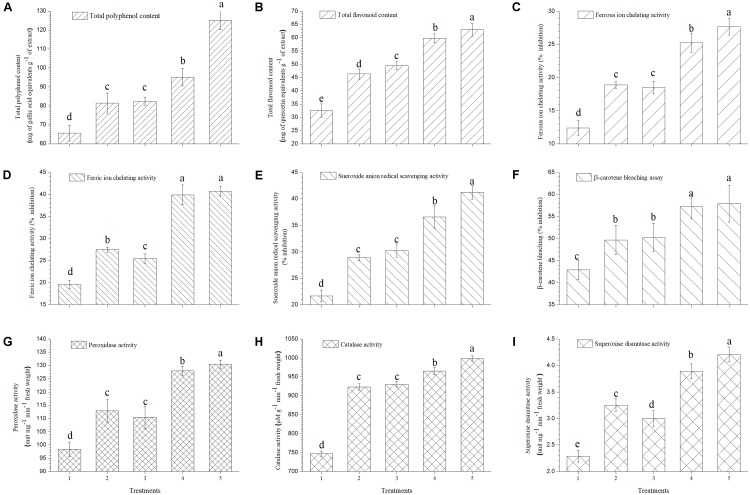
Effect of solid state cultivated *T. harzianum* UBSTH-501 on antioxidant enzymatic activities in ripe tomato fruits at harvest **(A)** Total polyphenol content, **(B)** total flavonoid content, **(C)** ferrous ion chelating activity, **(D)** ferric ion chelating activity, **(E)** superoxide anion radical scavenging activity, **(F)** β-carotene bleaching assay, **(G)** peroxidase activity, **(H)** catalase activity, and **(I)** superoxide dismutase activity. Treatments: 1- Control (untreated), 2- Fresh SMS, 3- Earthworm grazed SMS, 4- *T. harzianum* UBSTH-501 biofortified SMS, and 5- Earthworm grazed and *T. harzianum* UBSTH-501 biofortified SMS. Data are means (*n* = 5) and vertical bars represent standard error of means. Different letters above columns show significant difference in randomized block design test at *p* < 0.05 under Duncan’s multiple range test.

It was observed that total polyphenol and flavonoid content of ripe tomato fruits increased significantly in plants treated with EGTHB-SMS (125.19 mg of gallic acid equivalents g^−1^ of extract and 62.97 mg of quercetin equivalents g^−1^ of extract) as compared to the plants treated with only THB-SMS (95.23 mg of gallic acid equivalents g^−1^ of extract and 59.82 mg of quercetin equivalents g^−1^ of extract), EG-SMS (82.50 mg of gallic acid equivalents g^−1^ of extract and 49.52 mg of quercetin equivalents g^−1^ of extract) and fresh SMS (81.55 mg of gallic acid equivalents g^−1^ of extract and 46.33 mg of quercetin equivalents g^−1^ of extract). Minimum polyphenol and flavonoid content was recorded in ripe fruits obtained from control plants (65.66 mg of gallic acid equivalents g^−1^ of extract and 32.59 mg of quercetin equivalents g^−1^ of extract) (**Figures 6A,B**, respectively).

### Ferrous and Ferric Ion Chelating Activity

In the current study, further investigations were done on the role of the ripe tomato fruit extract on Fe^2+^ and Fe^3+^chelation, because these are the most effective pro-oxidants that are present in the food system (Halliwell et al., 1987). Maximum chelating effects on Fe^2+^ and Fe^3+^ ions (27.65 and 40.70%, respectively) were observed in the extracts of ripe fruits from plants treated with EGTHB-SMS followed by THB-SMS, EG-SMS and fresh SMS. However, minimum Fe^2+^ and Fe^3+^ ions chelation (12.40 and 19.65%, respectively) was recorded in the extracts of ripe fruits obtained from control plants at 10 mg ml^−1^ under nethouse conditions (**Figures 6C,D**).

### Effects on Superoxide Anion Radical Scavenging Activity and β-Carotene Bleaching Assay

The extract of fruits from plants treated with EGTHB-SMS was found to be the most potent superoxide scavenger (41.23%) compared to that of fruits from only THB-SMS (36.59%), EG-SMS (30.25%), fresh SMS (28.92%) and control (21.67%) plants (**Figure 6E**).

Auto-oxidation of β-carotene (β-carotene chelating assay) was used to evaluate the antioxidant activity of the ripe tomato fruit extracts. Results showed that extract taken from plants treated with EGTHB-SMS exhibited higher antioxidant activities (57.89%) compared to that from plants treated with THB-SMS (57.32%), EG-SMS (50.22%) and fresh SMS (49.67%), whereas extract of fruits from control plants was found to be least antioxidative (42.91%) on the selected parameter (**Figure 6F**).

### Antioxidant Enzymatic Activity

Earthworm grazed, *T. harzianum* biofortified SMS treated tomato exhibited higher antioxidant activities. The results exhibited more or less similar pattern for antioxidant enzymes activity in ripe tomato fruit extract as recorded for mineral content (**Figures 6G–I**). **Figure 6G** reflects higher peroxidase activity in ripe fruits from plants treated with EGTHB-SMS (130.49 unit mg^−1^ min^−1^ fresh wt.) compared to other treatments. As revealed in **Figure 6H**, the activity of catalase showed similar behavior, responding significantly to the different treatments. The superoxide dismutase (SOD) activity was highest in ripe fruits taken from plants treated with EGTHB-SMS (4.21 unit mg^−1^ min^−1^ fresh wt.) compared to plants treated with only THB-SMS (3.89 unit mg^−1^ min^−1^ fresh wt.), EG-SMS (3.00 unit mg^−1^ min^−1^ fresh wt.) and fresh SMS (3.25 unit mg^−1^ min^−1^ fresh wt.). However, least SOD activity was recorded in the extract of fruits taken from control plants grown under nethouse conditions (**Figure 6I**).

### Effect on Fruit Yield

**FIGURE 7:**
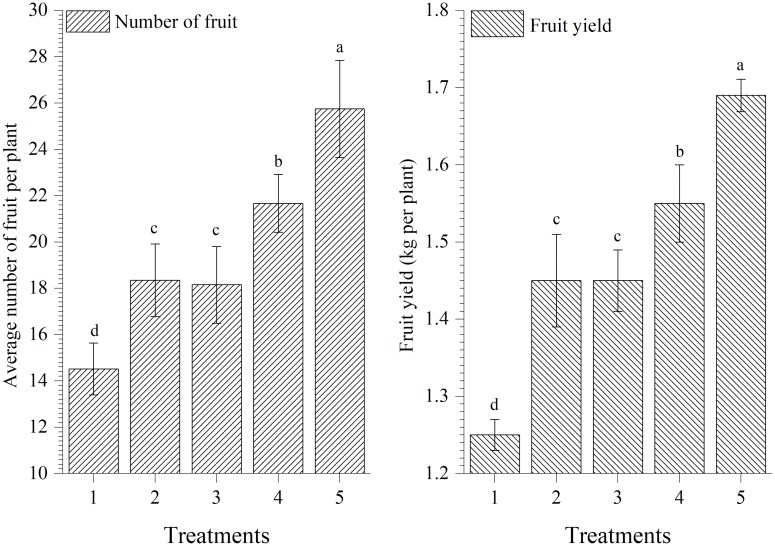
Effect of solid state cultivated *T. harzianum* UBSTH-501 on number of fruits per plant and yield of tomato at harvest. Treatments: 1- Control (untreated), 2- Fresh SMS, 3- Earthworm grazed SMS, 4- *T. harzianum* UBSTH-501 biofortified SMS, and 5- Earthworm grazed and *T. harzianum* UBSTH-501 biofortified SMS. Data are means (*n* = 5) and vertical bars represent standard error of means. Different letters above columns show significant difference in randomized block design test at *p* < 0.05 under Duncan’s multiple range test.

Results showed that plants treated with EGTHB-SMS produced significantly higher number of fruits per plants and yield (25.75 and 1.69 kg/plant, respectively) as compared to other treatments including plants treated with only THB-SMS (21.67 and 1.55 kg/plant, respectively), EG-SMS (18.15 and 1.45 kg/plant, respectively), fresh SMS (18.35 and 1.45 kg/plant, respectively) and control (14.52 and 1.25 kg/plant, respectively) under nethouse conditions after final harvest. However, lowest fruit yield was recorded in untreated control plants (**Figure 7**).

### Effect of Bio-Fortified SMS on Physico-Biochemical Properties of Soil

Results showed that amendment of fresh and biofortified SMS significantly affect the biochemical properties of the soil (**Table 3**). A significant increase in the soil organic carbon, organic matter and available macronutrients (N, P, K, and S) was recorded in the soil amended with earthworm grazed-*T. harzianum* biofortified SMS as compared to other treatments and initial value as presented in **Table 1**. Further, maximum available Fe, Mn, Zn, and Mo was recorded in the soil treated with earthworm grazed-*T. harzianum* biofortified SMS other treatments (**Table 3**). However, minimum CaCO_3_ was found in the soil amended with earthworm grazed-*T. harzianum* biofortified SMS.

**Table 3:** Effect of earthworm grazed and *T. harzianum* bio-fortified spent mushroom substrate on the physico-biochemical properties of post-harvested soil.

S.N.	Soil properties	Fresh SMS	*E. fetida* grazed SMS	*T. harzianum* UBSTH-501 fortified SMS	*E. fetida* grazed and *T. harzianum* UBSTH-501 fortified SMS
(1)	pH	7.2^b^	7.3^a^	7.3^a^	7.2^b^
(2)	EC (dSm^−1^)	0.89^b^	0.90^b^	0.95^a^	0.96^a^
(3)	Organic carbon (g kg^−1^)	5.66^d^	5.75^c^	5.82^b^	5.96^a^
(4)	Organic matter (g kg^−1^)	9.56^a^	10.05^a^	9.85^a^	10.33^a^
(5)	Bulk density (g cm^−3^)	1.38^a^	1.39^a^	1.37^a^	1.38^a^
(6)	Particle density (g cm^−3^)	2.48^b^	2.53^a^	2.50^b^	2.55^a^
(7)	CaCO_3_ (%)	6.75^a^	6.84^a^	6.72^a^	6.58^b^
(8)	**Available Macronutrients (kg ha^−1^)**				
(i)	N	195.60^c^	196.72^c^	198.35^b^	201.15^a^
(ii)	P	29.70^d^	30.95^c^	32.55^b^	35.19^a^
(iii)	K	152.33^c^	155.75^b^	153.82^c^	158.69^a^
(iv)	S	7.90^d^	8.25^c^	8.50^b^	8.96^a^
(9)	**Available micronutrient (mg kg^−1^)**				
(i)	Fe	18.90^c^	20.65^b^	20.25^b^	22.33^a^
(ii)	Mn	2.10^b^	2.66^a^	2.45^a^	2.50^a^
(iii)	Cu	1.10^b^	0.98^c^	1.25^a^	1.15^b^
(iv)	Zn	0.69^c^	0.69^c^	0.71^b^	0.76^a^
(v)	B	0.08^c^	0.10^b^	0.12^a^	0.09^b^
(vi)	Mo	0.05^c^	0.05^c^	0.06^b^	0.07^a^

*EC represents Electrical Conductivity, data are means (n = 3), data with different letters show significant difference in row data in Complete Randomized Design at p < 0.05 under Duncan’s multiple range test.*

## Discussion

Natural antioxidants and their role of in human health maintenance is a complex process and understanding these complex processes is the key to recognize cell responses against damage by free radicals. Free radicals (syn. reactive oxygen species-ROS) are produced in the cells as a by-product of normal metabolism and/or as a result of biotic and abiotic stresses (Sardesai, 1995). A promineprominent role of antioxidants in the reduction of ROS in the stressed tissues has already been established and reported by several workers (Halliwell, 1996; Devasagayam et al., 2004; Singh et al., 2010). ROS/FRs are normally neutralized by efficient systems in the body itself that include the antioxidant enzymes (peroxidase, catalase, SOD and glutathione peroxidase) and the nutrient-derived antioxidant small molecules such as lycopene, vitamin A, vitamin E, vitamin C, β-carotenes, polyphenolics, flavonoids, glutathione, etc. (Sardesai, 1995; Halliwell, 1996, 1997; Devasagayam et al., 2004). Since ancient era, fresh fruits, vegetables and herbal medicines have been the rich source of antioxidants in traditional human diet that protect tissues from the damage caused by free radicals (Sardesai, 1995; Sen and Chakraborty, 2011). In the present study, it has been demonstrated that application of *T. harzianum* biofortified SMS modulates accumulation of natural antioxidants and bio-fortification of mineral nutrients in tomato (*S.* *lycopersicum*) fruits rendering them more valuable as a food. Tomato constitutes an important source of antioxidants in Indian diet (George et al., 2004). The antioxidant activity of ripe tomato fruits has been tested using a wide variety of methods. Antioxidant activity is the capacity to prevent auto-oxidation of FR-mediated oxidation of the substrates when present at low concentration (Halliwell, 1992; Deepa et al., 2006; Bhanja et al., 2009). All these assays have been frequently used to assess antioxidant activity in the substrates (Deepa et al., 2006; Bhanja et al., 2009; Singh et al., 2010). Results from the present study involving earthworm grazing and *T. harzianum* biofortification of fresh SMS increases its physico-chemical and nutritional properties to significant extents. The nutritional quality of fresh SMS was even higher when the fresh SMS was co-inoculated with *T. harzianum* and earthworm in an additive manner as compared to only *T. harzianum* biofortified and earthworm grazed SMS. Results showed that optimized composting system with appropriate microorganisms and earthworm, *E. fetida* proved highly efficient in transforming SMS into odorless, porous and homogenized biofortified SMS. The improvement in nutritional quality of SMS may be attributed to accelerated mineralisation of the organic matter by *T. harzianum* and further enrichment by the earthworm feeding of biofortified SMS (Jordan et al., 2008). Further, several reports showed that earthworm grazing enhances physical property of the organic waste (Lalander et al., 2015). Recycling of these organics in agriculture after bio-fortification and earthworm grazing has several benefits such as improving soil carbon content and sustaining soil health that eventually lead to better crop produce and enhanced productivity (Lau et al., 2003; Jordan et al., 2008; Jonathan et al., 2011). In the current study, it was observed that application of EGTHB-SMS significantly increased the total carbohydrate, soluble sugar, proteins and lutein zeaxanthin in ripe tomato extract. Carbohydrate, sugars and proteins are the primary building blocks for any living cell (Singh et al., 2009a). The biofortified SMS carries substantial amount of essential plant nutrients, organics and enzymes which, on one hand, are useful for the better crop growth leading to improvement in the yield and returns thereby and on the other, help improve the nutritional quality of the produce (Lau et al., 2003; Jordan et al., 2008; Jonathan et al., 2011; Lalander et al., 2015).

Further application of biofortified SMS increases the antioxidant compounds namely lycopene and β-carotene in the ripe tomato fruits. There is an increase in concentration of β-carotene as the lycopene content increases, but the degree of increase is not similar. In ripe fresh tomato fruits, significant positive correlation has been found between lycopene and β-carotene content. Tomato lycopene and β-carotene have more bioactivity and bioavailability and also β-carotene is the precursor of vitamin A. Significant variation in concentrations of these compounds in different treatments might be due to the effect of solid-state cultivated *T. harzianum* and bioactive compounds present in the biofortified SMS. Organically cultivated fruits and vegetables generally tend to have higher concentrations of various essential nutrients like iron, phosphorous, potassium, vitamin C and several antioxidants compared to their conventionally grown counterparts (Barron, 2010; Montalba et al., 2010). The present investigation also validates the positive effects of *T. harzianum* enriched SMS on yield and quality of tomato fruits. While investigating the impact of nitrogen and bioinoculant *Bacillus licheniformis*, Ochoa-Velasco et al. (2016) have reported a positive effect of application of *B. licheniformis* on vitamin C and lycopene content in tomato. Similarly, enhancement in quality parameters of tomato due to the application of bio-augmented compost with effective microorganisms was reported by Verma et al. (2015). Currently, increased attention is being paid to evaluate the role of antioxidants in human diet and health (Singh et al., 2013c). Increase in lycopene, β-carotene, ascorbic acid and other antioxidant compounds in tomato under the influence of *T. harzianum* could help in improving dietary antioxidants and lowering cholesterol and oxidative burst effects, thereby aiding the prevention of several chronic diseases (Singh et al., 2013c) by better scavenging/quenching activities in the body (Bhanja et al., 2009; Singh et al., 2009a).

Moreover, lipid peroxidation by ROS/FRs may lead to the formation of toxic by-products/compounds such as malondialdehyde and 4-hydroxynonenal which can attack on membrane, DNA, inducing mutagenicity and carcinogenicity (Nautiyal et al., 2008; Singh et al., 2009a; Ademoyegun et al., 2011). The ability of any external agent to inhibit the oxidation process is often used to evaluate its antioxidant activity. In the present investigation, we used rat liver homogenate assay to investigate, whether the fresh extract of ripe tomato was able to protect the lipids from oxidation provoked by FeSO_4_-based HO^⋅^. In the light of the above findings, the most probable reason for fresh tomato extracts as FRs and/or ROS-scavengers might be the enhanced concentration of antioxidant compounds in the extract that have been reported to inhibit lipid peroxidation and protein oxidation by quenching FRs (Yang et al., 2000; Lee et al., 2008; Singh et al., 2009b, 2010). On the other hand, results also register the fact that plants treated with EGTHB-SMS accumulate more isoflavonoids and phenolics in the ripe tomato fruits. These are also powerful protecting agents against the lethal effects of oxidative damage and protect macromolecules by chelating redox-active transition metal ions (Bhanja et al., 2009; Singh et al., 2010). The present investigation indicates synergistic action of phenolic compounds in scavenging ROS, repairing protein and lipid peroxidation and metal chelation (Prakash et al., 2007a; Lee et al., 2008; Moktan et al., 2008; Singh et al., 2010).

The antioxidant efficiency of phenolic compounds has been related to the number of OH^⋅^ groups in their structures and also to their hydrogen radical donating abilities (Lemanska et al., 2001; Altunkaya et al., 2009). This observation is in agreement with the findings of other investigators (Lin et al., 2006; Lee et al., 2008; Singh et al., 2010). Higher polyphenolics in the extract might be due to *T. harzianum*-mediated formation or mobilization of free phenolic and flavonoid molecules in the tomato fruits from site of synthesis (Singh et al., 2010; Ahmad et al., 2016; Altunkaya et al., 2016). We further investigated the role of the ripe tomato fruit extract on Fe^2+^ and Fe^3+^chelation, because these are the most effective pro-oxidants that are present in the food system (Halliwell et al., 1987). The results also reveal that the tomato extracts showed a marked capacity for iron binding which might be helping in the lipid peroxidation and protein protection under stressed condition. The reducing capacity of a compound Fe^3+^ complex to the ferrous form may serve as a significant indicator of its antioxidant capacity (Roy and Rice-Evans, 1994). The existence of reductions is the key of the reducing power, which exhibit their antioxidant activities by breaking the free radical chain by donating a hydrogen atom (Schally and Nagy, 1999). The reduction of the Fe^3+^/ferric cyanide complex to the ferrous form occurs due to the presence of resultants in the solution. Chelating agents have been reported to be effective as secondary antioxidants as they reduce the redox potential, thereby stabilizing the oxidized form of the metal ions (Halliwell et al., 1987; Prakash et al., 2007b; Singh et al., 2009a, 2010). Superoxide ions directly and/or indirectly initiate oxidation of biomolecules such as nucleic acids, proteins, lipids and carbohydrates as a result of superoxide and hydrogen peroxide serving as precursors of singlet oxygen and HO^⋅^ (Singh et al., 2010). The present study also revealed that high flavonoid content in the extract leads to scavenging of superoxide radicals. This result is in agreement with the earlier findings of Singh et al. (2009a). Our results also indicate better O_2_^⋅−^ scavenging abilities in the extracts of plants treated with EGTHB-SMS than the control, and it might be due to formation of a glycones from glycosides of total phenolic and flavonoid and activation of superoxide dismutase (SOD) activity in the tomato fruits from *T. harzianum* treated plants. Furthermore, tomato is reported to contain sufficient amount of polyphenols (Singh et al., 2010, 2013b; Altunkaya et al., 2016), which are naturally occurring antioxidants, that possess an O_2_^⋅−^ scavenging effect similar to flavonoids (Singh et al., 2010; Ahmad et al., 2016).

The antioxidant activity assayed was the ability to inhibit the peroxidation of lipids. The higher antioxidant activity of extracts might be due to the inhibition of chain reaction, decomposing peroxides, preventing continued hydrogen abstraction and also attributed to the presence of antioxidant enzymes and phytochemicals, such as phenolics and isoflavones, ascorbic acid etc. (Singh et al., 2010). Our findings demonstrate a direct correlation between antioxidant activities and levels of enzymatic activity and are in agreement with the findings of some previous studies (Yang et al., 2008; Gao et al., 2010; Singh et al., 2013b,c). Further, the increase in the tomato yield is directly related to the higher nutritional content, enzymes and other plant growth promoting substrates present in the EGTHB-SMS. Furthermore, soil physico-biochemical properties were significantly affected by the application of biofortified SMS. Previous studies showed that application of organic matter significantly increases the soil biological properties (Sahu, 2011; Singh et al., 2013a; Lim et al., 2015). However, the available macro- and micronutrients in vermicompost are generally higher than in traditional compost produced from the same raw material (Lim et al., 2015). The biofortification further increases the quality of the compost and ultimately, influences the plant growth and quality of end products (Pramanik et al., 2007; Singh et al., 2008, 2013a). Additional indirect mechanisms include enhancement in the population of other beneficial microbes in the rhizosphere, enhanced nutrient use efficiency, and modulation of host physio-biological pathways leading to better plant growth and productivity (Ahlawat et al., 2006).

## Conclusion

The demand for organically grown fruits and vegetables has been growing steadily over the past few decades due to the awareness developed among people for a healthy lifestyle. Addition of organic manure and bioinoculants enhances the nutrient mobilization by the plants and helps them defend against various stresses by enhanced production of antioxidant enzymes and phenolics. The study describes the multifarious effects of EGTHB-SMS in tomato plants that significantly increase various attributes in the tomato fruits. Further, it was noted that EGTHB-SMS was found to be more potential in enhancing natural antioxidants, mineral nutrients and enzymes in the fruits. Encouraging results were also obtained with increase in nutritional quality of ripe tomato fruits (mineral nutrients and natural antioxidant properties) modulated by the application of EGTHB-SMS. Results of the investigation suggest that application of EGTHB-SMS in tomato not only helps in increasing yield and enhancing the nutritional value of ripe fruits but also reduces the risk of environmental pollution caused by piling and rather unsafe disposal of SMS.

## Author Contributions

UBS, DM, JPR, BKS, AKS, RC, MCM, and DP were involved in conceiving the idea and designing the work. UBS, DM, WK, SS, NK, MI, MCM, and RC conducted the actual research work. UBS, DM, WK, SS, NK, JPR, BKS, AKS, RC, MCM, and DP wrote the paper. However, J-WO contributed to the general designing and also in revising the MS during the review process. All authors were contributed to interpretation of results and discussion and approved the final version of the manuscript.

## Conflict of Interest Statement

The authors declare that the research was conducted in the absence of any commercial or financial relationships that could be construed as a potential conflict of interest.
